# Understanding Enterovirus D68-Induced Neurologic Disease: A Basic Science Review

**DOI:** 10.3390/v11090821

**Published:** 2019-09-04

**Authors:** Alison M. Hixon, Joshua Frost, Michael J. Rudy, Kevin Messacar, Penny Clarke, Kenneth L. Tyler

**Affiliations:** 1 Medical Scientist Training Program, University of Colorado School of Medicine, Aurora, CO 80045, USA; 2 Department of Immunology & Microbiology, University of Colorado School of Medicine, Aurora, CO 80045, USA; 3 Department of Neurology, University of Colorado School of Medicine, Aurora, CO 80045, USA; 4 Hospital Medicine and Pediatric Infectious Disease Sections, Department of Pediatrics, University of Colorado, Aurora, CO 80045, USA; 5 Children’s Hospital Colorado, Aurora, CO 80045, USA; 6 Division of Infectious Disease, Department of Medicine, University of Colorado School of Medicine, Aurora, CO 80045, USA; 7 Neurology Service, Rocky Mountain VA Medical Center, Aurora, CO 80045, USA

**Keywords:** enterovirus D68, acute flaccid myelitis, paralysis, neuropathogenesis, mouse models, experimental models

## Abstract

In 2014, the United States (US) experienced an unprecedented epidemic of enterovirus D68 (EV-D68)-induced respiratory disease that was temporally associated with the emergence of acute flaccid myelitis (AFM), a paralytic disease occurring predominantly in children, that has a striking resemblance to poliomyelitis. Although a definitive causal link between EV-D68 infection and AFM has not been unequivocally established, rapidly accumulating clinical, immunological, and epidemiological evidence points to EV-D68 as the major causative agent of recent seasonal childhood AFM outbreaks in the US. This review summarizes evidence, gained from in vivo and in vitro models of EV-D68-induced disease, which demonstrates that contemporary EV-D68 strains isolated during and since the 2014 outbreak differ from historical EV-D68 in several factors influencing neurovirulence, including their genomic sequence, their receptor utilization, their ability to infect neurons, and their neuropathogenicity in mice. These findings provide biological plausibility that EV-D68 is a causal agent of AFM and provide important experimental models for studies of pathogenesis and treatment that are likely to be difficult or impossible in humans.

## 1. Introduction

Enteroviruses (EVs) are classified into four groups (polioviruses, Coxsackie A viruses, Coxsackie B viruses, and echoviruses) and fifteen species, including four human EVs (A–D), 8 animal EVs (E–L), and 3 rhinoviruses (A–C). Several EVs cause neurologic disease in humans. Historically, the most well-known and most intensively investigated of the neuropathogenic EVs are the polioviruses. Due to decades of intensive vaccination efforts, however, paralytic poliomyelitis has been nearly eliminated as a clinical disease and cases are largely restricted to three countries - Pakistan, Afghanistan, and Nigeria [1]. In contrast, the reported incidence of neurologic infections by non-polio enteroviruses (NPEVs), including EV-A71, coxsackievirus A16 (CV-A16), and EV-D70, has increased globally in recent years [2]. 

One NPEV of current clinical and scientific interest is EV-D68. In 2014, this previously rare cause of pediatric respiratory disease re-emerged, resulting in an epidemic of pediatric respiratory disease in the United States (US) that was associated with a significant increase in cases of a polio-like paralytic syndrome referred to as acute flaccid myelitis (AFM). Additional cases of EV-D68-associated respiratory disease and AFM were subsequently recognized all over the world. Current research efforts are underway to understand the neuropathogenic potential of both historic and modern EV-D68 viral isolates. The goal of this review is to summarize recent basic research findings investigating the association between EV-D68 and neurologic disease. After summarizing the emergence of EV-D68 as a human pathogen, we will focus on three major areas of basic science research that have relevance for EV-D68 neurovirulence: (1) genetic analysis of viral isolates, (2) in vivo models of EV-D68 central nervous system (CNS) disease, and (3) in vitro studies of EV-D68 tropism and life cycle. This review will also highlight important gaps in our understanding of EV-D68 CNS pathology that future studies will need to address in order to develop a complete understanding of this re-emerging pathogen. 

## 2. Enterovirus D68 (EV-D68): A Re-Emerging Human Pathogen

EV-D68 was first identified in 1962 after it was isolated from oropharyngeal swabs taken from four children in California who had pneumonia and bronchiolitis. The four individual viral isolates from these children were named “Fermon”, “Rhyne”, “Franklin”, and “Robinson” [3]. These viral isolates were collectively referred to as the “Fermon virus,” after the first isolate recovered [3]. Basic assays identified the Fermon virus as an RNA virus that had chemical and biological characteristics consistent with EVs [3]. Later advancements in molecular biology and genetics led to the reclassification of the Fermon virus as enterovirus, species D, serotype 68 (EV-D68) [4]. 

Between the 1970s and 2005, EV-D68 was a rarely identified cause of human respiratory disease, and passive surveillance by the CDC National Enterovirus Surveillance System (NESS) recorded only 26 EV-D68 positive samples during this period [5]. This is likely an underrepresentation of the prevalence of EV-D68 associated disease as serotyping methods for EV-D68 were not widely available and testing of respiratory specimens for EVs was uncommon. However, the paucity of EV-D68 samples suggests that this virus was not likely a cause of widespread disease. Little further scientific work was performed on EV-D68 until the early 2000s. At this time, antibody cross-neutralization and partial genome sequencing were used to reclassify human rhinovirus 87 (HRV-87) as EV-D68 [6,7,8]. A detailed analysis of seventeen EV-D68 (and former HRV-87) isolates performed at the CDC demonstrated that EV-D68 was unique among the EVs [4]. Unlike conventional EVs, which are most commonly isolated from the stool and spread by fecal-oral transmission, all seventeen of the EV-D68 clinical specimens were isolated from the respiratory tract, suggesting spread by respiratory transmission [4]. Analysis of the isolates also showed that EV-D68 possessed biological characteristics more similar to the rhinoviruses than conventional EVs, including optimal replication in the cooler temperatures of the respiratory tract (33 °C) and acid lability [4]. 

In the years after 2005 and before 2014, health organizations around the world documented an increase in small EV-D68 respiratory outbreaks [9,10,11,12]. The majority of these cases occurred in young children, although some adult cases were reported. Clusters of children with disease often appeared seasonally in the months corresponding to late autumn and early winter in each hemisphere, a time window slightly later in the year than most other EVs (most common during the summer months). By the end of 2012, there were a total of 699 confirmed cases of EV-D68 worldwide, representing an enormous increase compared to the previous thirty years [9]. In the autumn of 2014, hospitals around the US experienced a sharp increase in pediatric respiratory disease admissions [13,14]. EV-D68 was identified as the major causal agent, with 1,395 cases of EV-D68 respiratory infections confirmed from August 2014-January 2015 by the CDC [15,16,17]. Studies of resource utilization and syndromic surveillance suggest that this number is likely to be a significant under-estimate of the overall size of the outbreak due to limited availability of EV-D68 assays and testing performed [18]. Over 1000 additional cases of EV-D68 respiratory disease were also confirmed in Canada, Europe, Asia, and South America [9]. Since 2014, there have been dozens of reports of EV-D68 circulation worldwide. A full summary of important clinical and epidemiological findings goes beyond the scope of this paper, but the global re-emergence of EV-D68 has been the topic of several excellent reviews [9,10,19,20,21,22,23]. 

Recently, European and US clinicians, as well as public health officials, have advocated for improved surveillance for EV-D68 [24,25,26,27]. The CDC has continued surveillance for EV-D68 respiratory disease using the NESS and the New Vaccine Surveillance Network (NVSN) [28,29,30,31]. Independent hospitals, such as Children’s Hospital Colorado, St. Louis Children’s Hospital, and Children’s Hospital of Philadelphia have also continued to perform surveillance for EV-D68 [32,33,34,35,36]. Data from these different networks and institutions suggest that EV-D68 has established a firm foothold as a human pathogen, with outbreaks occurring in a seasonal, biennial pattern within the US and regions of Europe since 2014 [37,38].

## 3. Enterovirus D68 (EV-D68)-Associated Acute Flaccid Myelitis (AFM)

A concerning and novel feature of the recent EV-D68 outbreaks is the association with cases of acute flaccid myelitis (AFM). Prior to 2014, EV-D68 was not considered a significant cause of neurologic disease. One case of flaccid paralysis in a young adult, where EV-D68 was found in the cerebrospinal fluid (CSF), was noted in the CDC NESS report covering 1970–2005, although details of this case have not been published [5]. There exists one additional report of EV-D68 found in a fatal case of meningo–myeloencephalitis in a 5-year-old child in the fall of 2008 [39]. This is more similar to recent AFM cases, which have occurred predominantly in children, with a mean age of onset between 3–8 years old [23]. The overwhelming majority of these affected children were immunocompetent and had completed standard childhood immunizations, including the inactivated poliovirus vaccine. The majority (80–90%) of children with AFM also experienced viral prodromal symptoms of fever and upper respiratory illness in the week prior to the onset of limb weakness [23]. The severity of respiratory disease, however, does not appear to correlate with the development of paralysis [27].

In distinction to poliomyelitis, EV-D68 associated AFM patients were more likely to be affected in upper limbs rather than lower limbs, consistent with injury to cervical rather than lumbar spinal motor neurons, although some children did develop widespread limb involvement, including quadriparesis [40,41,42]. Some affected children developed respiratory failure due to paralysis severe enough to require mechanical ventilation, and symptoms could include facial weakness and dysphagia consistent with brainstem (bulbar) involvement [40,41,42]. Magnetic resonance imaging (MRI) revealed longitudinally extensive T2 and FLAIR sequence hyperintense lesions in the grey matter of the spinal cord, localized to the anterior horns, and electromyography (EMG) was consistent with a lower motor neuron pattern of denervation [23,43,44,45]. Although some patients showed clinical improvement in the months after illness onset, the majority of patients had significant residual muscle weakness [40,46,47,48]. Fortunately, death has rarely been reported acutely in patients with AFM. 

In 2014, the CDC established diagnostic criteria for reporting cases of AFM, which were revised and adopted by the Council of State and Territorial Epidemiologists (CSTE) in June 2015, and subsequently revised in 2017 and 2019 [49]. Diagnosis of AFM for reporting purposes includes the acute onset of flaccid limb weakness plus confirmatory imaging evidence of spinal cord lesions spanning more than one vertebral segment in extent. The definition of AFM further specifies that spinal cord lesions be largely restricted to the gray matter, thus distinguishing AFM from acute flaccid paralysis (AFP), an umbrella term that encompasses any onset of acute limb weakness. The CSTE case definition notes that MRI abnormalities may be absent within the first 72 hours after presentation [49]. Earlier diagnostic criteria include a restriction to cases with age ≤18 years, although no age restriction is included in the current criteria. It is important to note that AFM as currently defined is a fairly broad and non-specific syndrome, and as such it is not surprising that cases have been associated with viruses other than EV-D68, including EV-A71, West Nile Virus (WNV), and adenoviruses. WNV appears to be a particularly notable cause of AFM occurring in adults, but this syndrome appears distinct from childhood AFM. In addition, recent descriptions of AFM cases associated with EV-A71 suggest that these cases have distinct clinical presentations and a different outcome from those associated with EV-D68 (Messacar et al., in review) [50]. Under the CSTE guidelines, 120 confirmed cases of AFM from 34 states were documented in 2014, 149 cases in 39 states (and the District of Columbia) in 2016, and 233 cases in 41 states in 2018 [32,33,34,35,51]. The strong temporal (biennial) and geographical correlation between these AFM cases and EV-D68 outbreaks implicates EV-D68 as a significant cause of AFM [27]. 

The gold standard of evidence for establishing a causal role for a specific virus in CNS infection is amplification of viral nucleic acid from CSF by polymerase chain reaction (PCR) or reverse transcription (RT)-PCR [52]. To date, only two CSF samples from US AFM patients since 2014 have been positive for EV-D68 by RT-PCR [53,54]. This absence of EV-D68-positive samples may be a result of the time of acquisition of CSF in the course of illness, as some neurotropic viruses lack detectable nucleic acid in the CSF at the time of clinical presentation, or may simply reflect absence of virus in the subarachnoid space despite its presence in selected parenchymal areas such as the spinal cord. It is important to note that EV-D68 RNA was detected in respiratory samples from AFM patients as the predominant pathogen in about half of the affected children tested within 1 week of prodromal symptom onset in 2014, with the rate of detection decreasing the later in the disease time course the patients were tested [42,55,56]. This suggests that failure of CSF detection of EV-D68 is unlikely to be due to technical aspects of the RT-PCR procedure, such as primer selection or design. It is also important to note that no other pathogen has been detected in respiratory samples with the frequency and consistency of EV-D68 [42,55,56]. These observations are consistent with results obtained from EV-D68-associated AFM cases at sites outside of the US as well [41]. The clinical and epidemiological evidence for establishing causality between EV-D68 and AFM has been summarized by two independent groups, and as such will not be covered in this review [26,27]. Instead we will focus on newly acquired evidence from in vitro and in vivo models of EV-D68 infection that demonstrate that recent, but not historic, strains of EV-D68 have gained the ability to infect neurons and to cause neurologic disease in mice. These experimental studies add “biological plausibility” to EV-D68 playing a direct causal role in AFM. 

## 4. Role of Viral Genetics in the Emergence of Enterovirus D68 (EV-D68) Neurovirulence

Like all EVs, EV-D68 belongs to Group IV of the Baltimore Classification System of viruses: single stranded, positive sense RNA viruses [57]. The EV-D68 genome consists of approximately 7400 RNA bases and contains the same canonical elements as other EV genomes – a 5’ untranslated region (UTR), a single open reading frame (ORF) containing all of the viral proteins, and a 3’ UTR [58]. The positive (+) sense RNA genome is directly infectious once released from its protein capsid or when transfected into cells. Using an internal ribosomal entry sequence (IRES) within the 5‘UTR, the viral RNA hijacks the host cell ribosome to begin immediate viral protein translation [58]. For EV species D, the protein-coding region is contained within the ORF and is translated as a single continuous polyprotein [58,59]. Within the polyprotein there are two proteases, 2A and 3C, that self-cleave the polyprotein into its individual components – the capsid proteins and the non-structural proteins [58]. Each genome also encodes an RNA-dependent-RNA-polymerase, which works in tandem with the viral 3B (VPg) protein to copy the RNA template of the virus [58]. The other non-structural proteins have important roles in promoting replication and host immune system disruption.

During the initial global emergence of EV-D68 in the early 2000s, several independent groups performed phylogenetic analyses of EV-D68 isolates. Each group found an increase in EV-D68 genome diversity when compared to early isolates of EV-D68 [11,60,61,62,63,64,65,66,67,68,69]. Molecular clock analysis of EV-D68 isolates from Europe, Asia, Africa, and the US revealed that newly circulating EV-D68 strains had diverged at some point between 1962 and the early 1990s into three distinct clades – A, B, and C. Compared to the historical prototype Fermon and Rhyne strains, all of the modern EV-D68 isolates lost a 24 RNA base region in the 5’UTR between the IRES and the polyprotein ORF [12,61]. Smaller changes and deletions were also present between the three clades [12]. Studies of additional isolates since the 2014 EV-D68 outbreak has led to further division of clade B into sub-clades B1, B2, and B3 [55,70,71], and a new clade has been recognized, clade D, with subclade divisions D1 and D2 [72,73,74]. An updated EV-D68 phylogenetic tree, including the time and location of strain isolations, can be found at nextstrain.org.

The data on EV-D68 lineage diversification from sites around the world suggests that EV-D68 continues to experience rapid evolutionary change. It is unclear whether the increased transmission of EV-D68, as well its association with neurologic disease, are due to specific changes within the viral genome, or simply due to increased circulation of EV-D68 in immunologically naïve populations. The loss of the 24-base region of the 5’UTR could explain the recent rapid spread of EV-D68, as the 5’UTR can affect viral fitness by altering translation initiation and efficiency [75]. In addition, studies with polioviruses, EV-A71, and Zika virus have found that even a single nucleotide mutation in the 5’UTR can alter (attenuate or confer) neurovirulence [76,77,78].

Intriguingly, analysis of EV-D68 strains isolated from respiratory samples of AFM patients in 2014 found that all of these AFM patients had been carrying EV-D68 strains from a single lineage, sub-clade B1. Genome sequence comparisons between sub-clade B1 strains and other EV-D68 lineages identified five unique polyprotein coding changes within B1 strains that shared sequence homology with neurovirulent poliovirus, EV-A71, or EV-D70 [55]. These findings were independently confirmed in a recent analysis of EV-D68 sequences deposited into the Virus Pathogen Resource (ViPR) database [79]. This study found that EV-D68 sub-clade B1 strains had 21 nucleotide substitutions involving both coding and non-coding regions that differed significantly from non-clade B1 EV-D68 strains. Of these 21 substitutions, 12 of the substitutions shared sequence identity with neuropathogenic polioviruses, EV-A71, and EV-D70, but not the non-clade B1 EV-D68 strains [79]. This clustering of polymorphisms in B1 strains with sequence similarity to neuropathogenic EVs has led to the hypothesis that EV-D68 genetic changes could explain the recent association between AFM and EV-D68. 

Of the available cases, EV-D68-associated AFM in the US, Taiwan, Netherlands, Sweden, Spain, Italy, and Argentina in 2016 have all mapped to the sub-clade B3, which appears to have emerged from a common ancestor of sub-clade B1 [70,80,81,82,83,84,85,86]. A study of EV-D68 isolates from the Children’s Hospital of Philadelphia found that viral strains from 2016 and 2018 clustered in sub-clade B3, but unlike isolates from 2014, lacked homology with other neurotropic EVs [36]. In 2018 there was one case of AFM associated with EV-D68 sub-clade B3 and one associated with sub-clade D1 in France [87]. This was surprising as sub-clade D1 has not been previously implicated in neurologic disease in humans. Co-circulation of EV-D68 has been found to result in inter-clade recombination, which could explain these findings, or sub-clade D1 may be neuropathogenic but has not had wide enough circulation to be previously identified as such [71,74]. 

As noted previously, respiratory specimen collection earlier in the course of AFM increased EV-D68 detection. Rapid identification of EV-D68-associated AFM cases on the part of healthcare providers is essential not only for clinical diagnosis but for future research efforts. Collection of additional strains has the potential to further identify EV-D68 lineages or genetic sequences correlated with the development of AFM. Healthcare provider education as to the signs and symptoms of AFM is a public health priority. When cases of EV-D68-associated AFM are identified, efforts should be made to collect respiratory swabs, stool or rectal swab, serum, and CSF samples for viral testing and genome sequencing. Current instructions on NPEV sample collection within the US are available on the CDC website, and the European non-polio enterovirus network (ENPEN) has outlined similar guidelines for European surveillance efforts [88,89].

## 5. Enterovirus D68 (EV-D68)-Induced Central Nervous System (CNS) Disease in Mouse Models

### 5.1. Strain-Dependent Paralysis

The original 1967 characterization of EV-D68 failed to demonstrate neuropathogenicity of the four prototype EV-D68 isolates in mice [3]. For these experiments, the researchers serially passaged the four prototype EV-D68 strains in primary rhesus monkey-kidney (MK) cells and injected them intracerebrally into neonatal Swiss Webster (SW) pups. Isolates Fermon, Franklin, and Robinson caused no demonstrable disease, however, limb weakness and paralysis was noted in mice infected with Rhyne [3]. This paralysis was found to be the result of muscle infection (myositis), and no evidence of brain or spinal cord lesions were noted on neuropathological examination.

**Figure 1:**
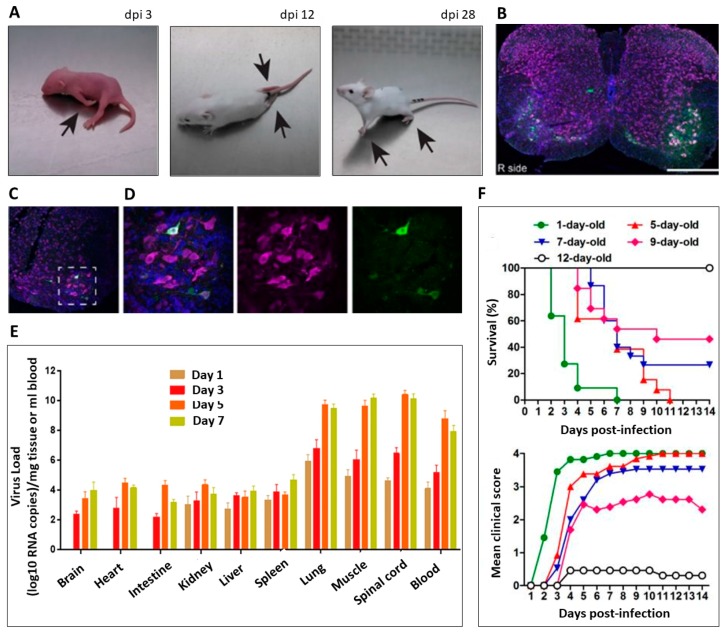
Contemporary strains of enterovirus D69 (EV-D68) cause paralysis in neonatal mice. (**A**) Examples of limb paralysis (arrows) in 3 different neonatal Swiss Webster (SW) mice following intracerebral (IC) injection of the contemporary EV-D68 strain MO/14-18947. Images are of mice at day post infection (dpi) 3, 12, and 28. (**B**) A cervical spinal cord section of a mouse at 100× original magnification following IC injection with EV-D68 MO/14-18947 that developed right forelimb paralysis on day 4 post-injection. Loss of motor neurons (green, labeled with choline acetyltransferase/ChAT) is observed in the right (“R side”) anterior horn, corresponding to the affected side. (**C**) 200× and (**D**) 600× images from a left anterior horn in an IC-injected mouse at 3 dpi before the onset of paralysis showing EV-D68 antigen in an intact cluster of motor neurons. The box in (**C**) represents the area imaged at 600× in (**D**). For all images, neurons (magenta) are labelled with NeuN, a general neuron marker, and nuclei (blue) are labeled with Hoechst 33342. Scale bars for 100× original magnification are 400 μm, 200× are 200 μm, 600× are 50 μm. (**E**) Tissue viral loads of 15296-virus (an EV-D68 strain produced by reverse engineering) in infected neonatal BALB/c mice. Samples of blood, brain, heart, intestine, kidney, liver, lung, muscle, spinal cord, and spleen were collected from infected mice following i.p. challenge with 5 LD50 of 15296-virus at 1, 3, 5 and 7 dpi (*n* = 3 per time point) or control mice given Dulbecco’s modified eagle medium (DMEM) at 0 days post-infection. Viral load was measured by real-time PCR, and the results represent the mean viral load ± SD. (**F**) Age dependence of EV-D68-induced disease and mortality. ICR mice (*n* = 11–15/group were intraperitoneally (IP) injected with 2.0 × 10^6^ TCID50 of US/MO/14-18947 per mouse at 1, 5, 7, 9, or 12 days of age. Survival and clinical score were then monitored and recorded daily. Clinical scores were graded as follows: 0, healthy; 1, lethargy and reduced mobility; 2, limb weakness; 3, limb paralysis; 4, death. Images taken from Hixon et al., 2017 (**A**–**D**), Sun et al., 2019 (**E**), and Zhang et al., 2018 (**F**).

In contrast to these prototype strains, contemporary EV-D68 isolates are neuropathogenic in mice. We found that several EV-D68 isolates from the 2014 US outbreak produce flaccid paralysis in neonatal SW mice following both intracerebral (IC) and intramuscular (IM) injection (examples of mice with paralysis following IC injection are shown in Figure 1A) [90]. In rare cases, paralysis was also seen after intranasal (IN) inoculation [90]. Regardless of the route of inoculation, paralysis corresponded to EV-D68 infection of the CNS and the loss of motor neurons in the anterior horns of the spinal cord (Figure 1B) corresponding to the segmental innervation of paralyzed limbs [90]. EV-like virions could be seen in motor neurons by electron microscopy and EV-D68 antigen was detectable in motor neurons (Figure 1C,D) [91]. In addition, Koch’s postulates are fulfilled in mice for the contemporary EV-D68 strain, MO/14-18947 [90]. 

As with the findings of the original EV-D68 paper, Fermon and Rhyne have not reliably produced neurologic disease in mice in any subsequent studies. Aside from the original strains isolated in 1967, there are few EV-D68 strains isolated prior to 2014 that are available for testing, but the ones tested thus far have also been non-paralytic in mice. More specifically, a recent testing of a single clade A member isolated in 2012 showed that that EV-D68 strain does not produce paralysis, and testing of strain VR1197, which was originally classified as HRV-87 Corn, revealed a similar result [92]. In contrast, the neuropathogenicity of contemporary EV-D68 strains has been replicated by other research groups in several different mouse models. Table 1 summarizes the paralytic and non-paralytic EV-D68 strains used in each study along with their clade and source. 

Data obtained from these mouse models suggests that neuroparalytic strains of EV-D68 may have only recently emerged, however there does not appear to be a clear division along clade or subclade lines between paralytic or non-paralytic isolates. As previously discussed, viral genome analyses have suggested that clade B1 strains have shifted towards increased sequence homology with other neurovirulent EVs, providing one hypothesis in explaining the appearance of AFM. However, data from the animal studies have shown that EV-D68 strains from clades B1, B2, and D1 possess the capacity to produce paralysis [90,93]. In addition, some contemporary strains isolated in 2014 do not cause paralysis. For example, clade B2 member CA/14-4231 is non-paralytic, while the closely related clade B2 member IL/14-18952 is highly paralytic [90]. These strains are 98% identical by genome sequence and possess only 11 coding differences between them. 

To our knowledge, all strains tested in mouse models have been respiratory isolates from individuals with EV-D68 respiratory infection without neurologic symptoms. Isolation of sufficient quantities of replication-capable EV-D68 virus from AFM patients, in either respiratory or CSF samples, has likely been hindered by the delayed presentation of the neurologic disease in comparison to the respiratory symptoms. It is currently unclear whether strains used in mouse research possess the same biologic properties (growth kinetics, tissue tropism, receptor utilization, etc.) as EV-D68 viral isolates from AFM patients. Thus, EV-D68 research would be advanced by the ability to quickly recognize and test clinical AFM cases. Improved detection methods would not only improve EV-D68 surveillance but would also aid efforts to focus EV-D68 studies on strains that are most prevalent or most pathogenic. Furthermore, there is a significant need for the generation of EV-D68 infectious clones and chimeras from available EV-D68 strains to systematically test polymorphisms or genome regions that confer neurovirulence in these mouse models. The development of these tools represents a top priority for future EV-D68 basic science research.

### 5.2. Enterovirus D68 (EV-D68) Tropism and Spread

Several other notable observations have been made in mouse models that are of significant interest in understanding the role of EV-D68 in the pathogenesis of AFM. First, EV-D68 exhibits strong tropism for spinal motor neurons in vivo, but not neurons in general [90,91]. We found that the most efficient route of inoculation to produce paralysis in mice is to inject directly into limb muscle. This results in rapid growth of virus in the muscle followed by growth in the spinal cord, but not in the brain [90]. In parallel with this observation, we found that even direct IC inoculation resulted in little or no growth of EV-D68 growth in brain tissue [90]. Similar results were also noted in two additional mouse studies by other research groups [93,94]. Following intraperitoneal (IP) infection of EV-D68, neonatal BALB/c mice developed paralysis that was associated with low levels of EV-D68 in all tissues examined, as well as blood, with the highest viral levels found in muscle, lungs, and spinal cord (Figure 1E) [93]. IP injection of EV-D68 did not induce paralysis in SW mice [90]. The difference in susceptibility of BALB/c and SW mice may be due to the fact that BALB/c mice are immunodeficient. EV-D68 also induced paralysis in neonatal Institute of Cancer Research (ICR) mice following IP infection with infection of the muscle and spinal cord tissue [94]. In these two models, virus levels in brain were not significantly elevated. The development of mouse models in multiple mouse strains has highlighted differences in susceptibility to EV-D68 infection [90]. These differences suggest that host factors and infection sites play a key role in the restriction or promotion of viral spread to the CNS. The ability to identify host genetic determinants of susceptibility to EV-D68-induced paralysis in each mouse strain could lead to a more complete understanding of this disease and novel mechanisms for drug targeting against viral spread.

The localized pattern of motor neuron involvement in mice suggests that EV-D68 infection travels directly, potentially via neural pathways, from the site of primary infection to the spinal cord. IM injection of paralytic EV-D68 in mice resulted in paralysis onset starting in in the injected limb [90,95]. Mice injected IC or inoculated IN were more likely have paralysis onset in their forelimbs rather than hindlimbs [90]. Interestingly, mouse studies performed in adult AG129 mice reported consistent paralysis onset in the hindlimb ipsilateral to the IP injection site [91,93,94]. This result is somewhat surprising, as IP injection is more akin to intravenous injections, where one might expect to find viremia and a more random distribution of affected limbs, as seen with neurotropic viruses such as reovirus [96]. It should be noted that the AG129 mice used in this study lack receptors for interferon (IFN) alpha, beta, and gamma allowing EV-D68 to induce paralytic disease in 10-day-old mice. Most neurotropic viruses show increased neurovirulence following direct IC as compared to peripheral inoculation. The fact that EV-D68 is more paralytogenic after peripheral, compared to IC, inoculation is unusual for neurotropic viruses and is consistent with a specific viral predilection for spinal motor neurons. As previously discussed, human AFM patients have lesions within the spinal cord and brain stem motor nuclei on MRI, but do not exhibit signs, symptoms, or laboratory findings of encephalitis. Human AFM patients are also more likely to have weakness involving upper limbs than lower limbs. Viremic spread from the respiratory tract to the spinal cord would not be expected to produce a preferential pattern of upper limb weakness, as all regions of the spinal cord would presumably be equally exposed to hematogenous virus. Studies comparing infection between mouse brains and spinal cord, ex vivo slice culture, or dissociated cell cultures could provide further insights into the nature of this strict pattern of neurotropism. 

### 5.3. Age-Dependent Paralysis

Similar to humans, mice exhibit age-dependent susceptibility to EV-D68-induced neurologic disease. One recent study demonstrated that injection of EV-D68 at progressively later post-natal time points results in reduced mortality and improved functionality scores in ICR mice following IP EV-D68 infection (Figure 1F) [93]. By post-natal day 12, mice were almost completely immune to development of EV-D68-induced paralysis [93]. We have noted similar results in both SW and C57/Bl6 mice following IC and IM injection (Tyler lab unpublished data). Another recent mouse study demonstrated that AG129 mice, (which lack receptors for α/β/γ –IFN - see above), have prolonged susceptibility to EV-D68 induced-paralysis, and develop severe AFM after IP infection at ten-days old [91]. Examination of compound muscle action potentials (CMAP), nerve conduction velocity (NCV), and hematoxylin-eosin (H&E) histochemistry revealed that AFM in AG129 mice is the result of both myositis and severe myelitis that persists up to 6 weeks post infection [91]. The extent to which immune maturation influences age-related susceptibility to EV-D68 associated AFM remains unclear. The current knowledge on EV-D68’s interaction with the innate immune system was recently summarized in a review by Sun et al., and continuing investigation into the IFN-regulated immune factors that restrict EV-D68 could help explain the age-related susceptibility to EV-D68-induced CNS infection [97]. 

### 5.4. Antibody-Mediated Protection

An antibody-mediated immune response following peripheral EV-D68 infection has been demonstrated in several mouse models (SW, ICR, and BALB/c). Immune sera from mice infected with paralytic EV-D68 as neonates has been shown to prevent paralysis by EV-D68 in immunologically naïve animals [90,93,94]. ICR and BALB/c dams inoculated with inactivated neurotropic EV-D68 prior to conception produced maternal neutralizing antibodies that prevented the development of paralysis in offspring challenged with paralytic EV-D68 [93,94]. Interestingly, inoculation of BALB/c dams with inactivated EV-D68 Fermon also conferred protection against a paralytic EV-D68 isolate (EV-D68 15296) [93]. We previously found that SW mice do not generate antibodies to EV-D68 Fermon after IM inoculation, likely due to the failure of this virus to replicate significantly in the muscle tissue of inoculated animals [90]. We also found that SW anti-EV-D68 immune sera generated against a single EV-D68 isolate (US/IL/14-18952) had reduced ability to neutralize other EV-D68 isolates in a manner dependent on phylogenetic relationship, with more distantly related isolates showing less neutralization. The differences in production of neutralizing antibodies between mouse strains could be explained by the difference in susceptibility to infection seen between SW and ICR/BALB/c mice. This difference further highlights the importance of understanding determinants of EV-D68 infection and immune response in different mouse strains. 

These mouse studies could hold promise for the eventual development of an EV-D68 vaccine. In pursuit of this goal, Dai et al. recently developed a virus-like particle (VLP) containing EV-D68 capsid proteins VP0, VP1, and VP3, that produced a strong neutralizing antibody response in 6–8- week-old ICR female mice [98]. Serum from these mice effectively neutralized two Clade A strains from the 2014 outbreak (US/MO/14-18947 and US/KY/14-18953), as well as the prototype Fermon strain [98]. These results are promising, although as AFM is still a very rare condition, with <1% of EV-D68 infections resulting in AFM, the number of people that would need to be treated with an EV-D68 vaccine to prevent a single case of AFM is estimated to be high (>1000). Many questions also remain about the efficacy of antibody-mediated immune protection against EV-D68 in the human population. A survey of human serum samples banked prior to the 2014 outbreak in Kansas City, MO showed universal seroprevalence of neutralizing anti-EV-D68 antibodies to both contemporary and historic EV-D68 isolates, although lower neutralizing titers were found in children age 2–5 years old [99]. Commercially available human intravenous immunoglobin (hIVIG) collected between June 2015 and January 2016 has been shown to protect against the development of paralysis in SW mouse pups after passive immunization [95,100]. Despite the high seroprevalence of broadly neutralizing antibodies, EV-D68 continues to circulate and cause disease. Future studies exploring the presence of neutralizing antibodies in specific tissue compartments, such as secreted IgA in the lung, may be more informative at predicting susceptibility to the development of respiratory infections and AFM across time in vulnerable populations. 

### 5.5. Anti-Viral Strategies for the Treatment of Enterovirus D68 (EV-D68) Infections

Historically, vaccination has been more effective than antiviral treatment in combating neurological disease resulting from EV (poliovirus and EV-A71) infections. To date, there are currently no effective antiviral drugs available for EV-D68, with current treatment primarily focused on supportive care. Within the US, a majority of AFM patients received intravenous immunoglobulin (IVIG) and high-dose steroid treatments during the 2014 outbreak, and a small percentage of patients received antiviral therapy [42]. Although a systematic analysis of treatment efficacies in 2014 is not available, no significant changes were noted in patients receiving these antiviral therapies [42]. However, IVIG treatment significantly reduces paralysis and EV-D68 viral load in SW mice [95]. These results are consistent with the high prevalence of neutralizing antibodies seen in human sera (see above), and although not officially recommended by the CDC, IVIG has been used to supplement supportive care in many AFM cases [42,100].

Antiviral therapies against EV-D68 are still being actively pursued with varying degrees of success. The anti-depressant Fluoxetine was tested as a potential treatment during early outbreaks due to its ability to inhibit EV-D68 in vitro but showed no apparent effect in clinical settings [101,102]. Furthermore, fluoxetine did not decrease viral load or disease severity in SW mice, while the steroid dexamethasone worsened disease in our SW mouse model [95]. One study assessed the capsid binding compounds pleconaril, vapendavir, and pirodavir for their ability to inhibit EV-D68 replication in vitro in HeLa cells [103]. The compounds were tested against ten 2014 isolates of EV-D68, and pleconaril was found to be the most effective of the three capsid binding inhibitors. The same study found that the 3C protease inhibitors rupintrivir and SG85 were also highly effective against all 10 EV-D68 strains, while the broad spectrum antiviral Favipiravir was not [103]. A subsequent study reported similar results, demonstrating that pleconaril and rupintrivir effectively inhibit a clade B1 strain of EV-D68 (US/KY/14-18593) in rhabdomyosarcoma (RD) cells [104]. These initial in vitro results may hold promise for the development of an antiviral therapy for EV-D68, but it remains to be determined if these compounds will have a similar inhibitory effect in vivo.

## 6. Enterovirus D68 (EV-D68) Neurotropism in Neuron Culture Models

### 6.1. Infection of Neurons

Recent studies in neuronal culture models have provided further evidence supporting the neurotropism of contemporary EV-D68 strains. Strains isolated during the 2014 outbreak have the ability to infect and replicate in a neuroblastoma-derived neuronal cell line (SH-SY5Y) as well as human postnatal cortical neuron cultures [92]. However, strains isolated prior to the 2014 outbreak could not grow in these neuronal cells [92]. In contrast, non-neuronal HeLa and A549 (lung carcinoma) cells supported infection of all EV-D68 strains tested. Differential cell entry as the primary factor for determining infectivity between contemporary and historic EV-D68 strains was supported using cell binding assays. In addition, transfection of historic strains into cells failed to sustain an active infection, as viral progeny produced following transfection were unable to produce a sustained infection beyond the first round of replication [92].

Two additional studies have identified differential receptor utilization as the key factor determining neurotropism. One study generated infectious clones of EV-D68 Fermon and EV-D94, another related species D enterovirus [101]. EV-D94 exhibited the ability to infect neuroblastoma cells and human primary neurons, while EV-D68 Fermon did not. A chimeric virus generated to encode the capsid of EV-D94, with the remaining regions derived from EV-D68 Fermon, lost the ability to infect neuronal cells, suggesting that regions outside the capsid may restrict neurotropism [101]. Finally, a recent study by our lab demonstrated that all strains of EV-D68 (contemporary and historic) have the ability to infect and undergo retrograde transport in human-derived induced-pluripotent stem cell (iPSC) spinal motor neurons grown in a microfluidic culture system in which the axons are spatially isolated from the motor neuron soma [102]. However, cleavage of sialic acid from the motor neuron axons by the enzyme neuraminidase completely prevented infection by historic (pre-2014) strains of EV-D68 but had no effect on infection by contemporary strains. 

Overall, the ability to infect neuronal cells corresponds to the ability to produce AFM-like disease in mouse models with a few critical exceptions. All contemporary EV-D68 strains tested so far appear to have the ability to infect neuronal cells, yet, some contemporary EV-D68 strains such as EV-D68 CA/14-4231 do not appear to possess the ability to produce paralysis in SW [90] or C57Bl/6 mice (Tyler lab unpublished data). These studies indicate the intrinsic ability to infect neurons is not the only required determinant for development of CNS disease. Examination of host factors that determine development of CNS disease represents an important area for future AFM research. 

### 6.2. Receptor Binding

Identifying the cellular receptors responsible for viral attachment and uncoating is a key step in understanding neuropathogenesis. Both sialic acid residues and ICAM-5 (telencephalin) have been identified as receptors capable of facilitating EV-D68 infection, although the biological significance of these receptors in vivo is unknown. Sialic acid is a nine-carbon monosaccharide that is most prevalent on cells in the respiratory tract but can serve as terminal modification to proteins and lipids in tissues throughout the body. These sugars are a common receptor for many viruses, particularly respiratory viruses such as influenza [103]. Consistent with EV-D68 respiratory pathology, a recent haploid genetic screen of the ancestral Fermon EV-D68 strain identified several genes involved in the biosynthesis, transport, and conjugation of sialic acid that were essential for infection [8,104]. More specifically, Fermon preferentially bound α2,6-linked sialic acid, which is the predominant linkage in the upper respiratory tract [105]. This sialic acid linkage binds to a narrow canyon in the Fermon capsid and is thought to displace the hydrophobic “pocket factor” normally responsible for stabilizing the capsid structure [105]. In contrast to prototype EV-D68 strains, recent studies on contemporary strains of EV-D68 have demonstrated that modern strains do not require sialic acid for cell entry and infection, indicating that some EV-D68 strains have the ability to bind non-sialylated receptors [104]. While these contemporary strains can still cause the hemagglutination of red blood cells, indicative of sialic acid binding, it is clear that sialic acid is not a required receptor for paralytic EV-D68 strains [104]. Sialic-acid independent neuronal entry may thus also contribute to the increased neuroinvasiveness of contemporary, compared to historic, EV-D68 strains. 

The neuron-specific intercellular adhesion molecule 5 (ICAM-5/telencephalin) has been identified as a possible receptor for both sialic acid-dependent and -independent EV-D68 viruses [106]. The ICAM family proteins are immunoglobulin-like transmembrane glycoproteins often found in the brain and lungs and include receptors for several enteroviruses including the poliovirus receptor, ICAM-1 [107,108]. Expression of ICAM-5 in Vero cells allowed EV-D68 to infect and replicate in this otherwise non-permissive cell type [106]. Furthermore, down-regulating ICAM-5 expression in permissive cell lines inhibited EV-D68 infection in vitro, as did a soluble ICAM-5-Fc fragment (serving as a decoy receptor) [106]. Although the ICAM-5 protein contains sialic acid residues, desialylated soluble ICAM-5, functioning as a decoy receptor, still inhibited EV-D68 infection, indicating that binding to ICAM-5 was occurring independent of its sialic acid moieties. 

The role of ICAM-5 in neuronal infection and entry remains unclear. The distribution and expression of ICAM-5 in human nervous tissues has been examined by western immunoblotting [101]. ICAM-5 was found to be consistently expressed in both pediatric and adult cortical tissue, but no ICAM-5 protein was detected in pediatric cervical spinal cord tissue [102]. Furthermore, ICAM-5 protein could not be detected in neonatal mouse spinal cords, despite obvious evidence of neuronal infection and injury [102]. EV-D68 has not been found to infect cortical tissue in either the mouse model or human patients, and instead appears to display a strong tropism for motor neurons found in the spinal cord. The lack of detectable ICAM-5 expression in spinal cords suggests that this receptor does not play a role in the development of AFM. Consistent with this idea, we recently demonstrated that in vitro ICAM-5 expression in human iPSC-derived motor neuron-like cells is restricted to the soma and dendrites of these cells and was not detectable by immunohistochemistry on the axon terminals where EV-D68 has been shown to be capable of entering neurons [102]. These studies do not exclude the possibility that another member of the immunoglobulin superfamily with structural similarity to ICAM-5 may serve as the actual EV-D68 receptor on neurons. Further research is clearly needed to definitively identify the receptor(s) responsible for mediating CNS infection by contemporary strains of EV-D68.

## 7. Conclusions

EV-D68 neurovirology is a burgeoning field with a new and growing research community. Recent work from multidisciplinary, international research teams has dramatically expanded our understanding of this virus in the five years since the 2014 outbreak. Improved surveillance and isolation of EV-D68 strains has revealed continuing viral diversification, including the identification of several genome polymorphisms that have been linked to neurovirulence. Mouse models of AFM have been developed that replicate several cardinal features of human disease. Neuronal culture models are making progress in understanding the mechanisms of viral neurotropism. These data provide biological plausibility for EV-D68 as a cause of neurologic disease. While research advances have improved our ability to study EV-D68, they have also revealed the complexity of EV-D68 neuropathogenesis. In this review, we have summarized the current state of basic science research on EV-D68 neurologic disease and have highlighted gaps in our understanding of EV-D68 pathogenesis. We hope that identification of these gaps, as well as discrepancies between models, stimulates new research questions. 

## Figures and Tables

**Table 1:** Paralytic and non-paralytic enterovirus D68 (EV-D68) strains.

	Strain	Clade	Route of Admin	Accession #	Source	Reference
**Paralytic**	MO/14-18947	B1	IC, IM, IN, IP	KM851225	2014, USA, Respiratory	Hixon et al. 2017 Zhang et al. 2018
	MO/14-18949	B1	IP	MH708882	2014, USA, Respiratory	Morrey et al. 2018
	CA/14-4232	B1	IC	KU844180	2014, USA, Respiratory	Hixon et al. 2017
	IL/14-18952	B2	IC, IM	KM851230	2014, USA, Respiratory	Hixon et al. 2017 PLoS Hixon et al. 2017 JID
	Beijing-R0132	B2	IP	KP240936	2014, China, Respiratory	Sun et al. 2019
	KY/14-18953	D1	IC, IP	KM851231	2014, USA, Respiratory	Hixon et al. 2017
**Non-paralytic**	CA/14-4231	B2	IC	KU844181	2014, USA, Respiratory	Hixon et al. 2017
	USA/N0051U5/2012	A1	IM	KT347280	2012, USA, Respiratory	Brown et al. 2019
	VR1197 *	Proto type	IM	KT725431	Respiratory	Brown et al. 2019
	Fermon	Proto type	IC, IM	AY426531	1962, USA, Respiratory	Scheible et al. 1967 Hixon et al. 2017 Zhang et al. 2018
	Rhyne	Proto type	IC, IM	KU844178	1962, USA, Respiratory	Scheible et al. 1967 Hixon et al. 2017
	Franklin	Proto type	IC		1962, USA, Respiratory	Scheible et al. 1967
	Robinson	Proto type	IC		1962, USA, Respiratory	Scheible et al. 1967

## References

[1] Racaniello V.R. (2006). One hundred years of poliovirus pathogenesis. Virology.

[2] Pons-Salort M., Parker E.P., Grassly N.C. (2015). The epidemiology of non-polio enteroviruses: Recent advances and outstanding questions. Curr. Opin. Infect. Dis..

[3] Schieble H.J., Fox V.L., Lennette E.H. (1967). A probable new human picornavirus associated with respiratory diseases. Am. J. Epidemiol..

[4] Oberste M.S., Maher K., Schnurr D., Flemister M.R., Lovchik J.C., Peters H., Sessions W., Kirk C., Chatterjee N., Fuller S. (2004). Enterovirus 68 is associated with respiratory illness and shares biological features with both the enteroviruses and the rhinoviruses. J. Gen. Virol..

[5] Khetsuriani N., LaMonte-Fowlkes A., Oberst S., Pallansch M.A. (2006). Centers for Disease Control and Prevention. Enterovirus surveillance—United States, 1970–2005. MMWR Surveill. Summ.

[6] Ishiko H., Miura R., Shimada Y., Hayashi A., Nakajima H., Yamazaki S., Takeda N. (2002). Human rhinovirus 87 identified as human enterovirus 68 by VP4-based molecular diagnosis. Intervirology.

[7] Savolainen C., Blomqvist S., Mulders M.N., Hovi T. (2002). Genetic clustering of all 102 human rhinovirus prototype strains: Serotype 87 is close to human enterovirus 70. J. Gen. Virol..

[8] Blomqvist S., Savolainen C., Råman L., Roivainen M., Hovi T. (2002). Human rhinovirus 87 and enterovirus 68 represent a unique serotype with rhinovirus and enterovirus features. J. Clin. Microbiol..

[9] Holm-Hansen C.C., Midgley S.E., Fischer T.K. (2016). Global emergence of enterovirus D68: A systematic review. Lancet Infect. Dis..

[10] Messacar K., Abzug M.J., Dominguez S.R. (2016). The Emergence of Enterovirus-D68. Microbiol. Spectr..

[11] Rahamat-Langendoen J., Riezebos-Brilman A., Borger R., van der Heide R., Brandenburg A., Schölvinck E., Niesters H.G. (2011). Upsurge of human enterovirus 68 infections in patients with severe respiratory tract infections. J. Clin. Virol..

[12] Tokarz R., Firth C., Madhi S.A., Howie S.R., Wu W., Sall A.A., Haq S., Briese T., Lipkin W.I. (2012). Worldwide emergence of multiple clades of enterovirus 68. J. Gen. Virol..

[13] Midgley C.M., Watson J.T., Nix W.A., Curns A.T., Rogers S.L., Brown B.A., Conover C., Dominguez S.R., Feikin D.R., Gray S. (2015). Severe respiratory illness associated with a nationwide outbreak of enterovirus D68 in the USA (2014): A descriptive epidemiological investigation. Lancet Respir. Med..

[14] Oermann C.M., Schuster J.E., Conners G.P., Newland J.G., Selvarangan R., Jackson M.A. (2015). Enterovirus d68. A focused review and clinical highlights from the 2014 U.S. Outbreak. Ann. Am. Thorac. Soc..

[15] Midgley C.M., Jackson M.A., Selvarangan R., Turabelidze G., Obringer E., Johnson D., Giles B.L., Patel A., Echols F., Oberste M.S. (2014). Severe respiratory illness associated with enterovirus D68—Missouri and Illinois, 2014. MMWR Morb. Mortal. Wkly. Rep..

[16] Centers for Disease Control and Prevention (2016). Enterovirus D68. http://www.cdc.gov/non-polio-enterovirus/about/ev-d68.html.

[17] Brown B.A., Nix W.A., Sheth M., Frace M., Oberste M.S. (2014). Seven Strains of Enterovirus D68 Detected in the United States during the 2014 Severe Respiratory Disease Outbreak. Genome Announc..

[18] Messacar K., Hawkins S.M., Baker J., Pearce K., Tong S., Dominguez S.R., Parker S. (2016). Resource Burden During the 2014 Enterovirus D68 Respiratory Disease Outbreak at Children’s Hospital Colorado: An Unexpected Strain. JAMA Pediatr..

[19] Cassidy H., Poelman R., Knoester M., Van Leer-Buter C.C., Niesters H.G.M. (2018). Enterovirus D68—The New Polio?. Front. Microbiol..

[20] Esposito S., Bosis S., Niesters H., Principi N. (2015). Enterovirus D68 Infection. Viruses.

[21] Imamura T., Oshitani H. (2015). Global reemergence of enterovirus D68 as an important pathogen for acute respiratory infections. Rev. Med. Virol..

[22] Messacar K., Abzug M.J., Dominguez S.R. (2016). 2014 outbreak of enterovirus D68 in North America. J. Med. Virol..

[23] Helfferich J., Knoester M., Van Leer-Buter C.C., Neuteboom R.F., Meiners L.C., Niesters H.G., Brouwer O.F. (2019). Acute flaccid myelitis and enterovirus D68: Lessons from the past and present. Eur. J. Pediatr..

[24] Domingo F.R., McMorris O., Mersereau T. (2016). Surveillance of the emerging enterovirus D68 in Canada: An evaluation. Can. Commun. Dis. Rep..

[25] Pariani E., Pellegrinelli L., Merlone A.D., Piralla A., Baldanti F., Binda S. (2017). Letter to the editor: Need for a European network for enterovirus D68 surveillance after detections of EV-D68 of the new B3 lineage in Sweden and Italy, 2016. Eurosurveillance.

[26] Dyda A., Stelzer-Braid S., Adam D., Chughtai A.A., MacIntyre C.R. (2018). The association between acute flaccid myelitis (AFM) and Enterovirus D68 (EV-D68)—What is the evidence for causation?. Eurosurveillance.

[27] Messacar K., Asturias E.J., Hixon A.M., Van Leer-Buter C., Niesters H.G., Tyler K.L., Abzug M.J., Dominguez S.R. (2018). Enterovirus D68 and acute flaccid myelitis-evaluating the evidence for causality. Lancet Infect. Dis..

[28] Kujawski S.A., Midgley C.M., Rha B., Lively J.Y., Nix W.A., Curns A.T., Payne D.C., Englund J.A., Boom J.A., Williams J.V. (2019). Enterovirus D68-Associated Acute Respiratory Illness—New Vaccine Surveillance Network, United States, July–October, 2017 and 2018. MMWR Morb. Mortal. Wkly. Rep..

[29] Center for Disease Control and Prevention New Vaccine Surveillance Network (NVSN). https://www.cdc.gov/surveillance/nvsn/index.html.

[30] Center for Disease Control and Prevention National Enterovirus Surveillance System (NESS). https://www.cdc.gov/surveillance/ness/index.html.

[31] Lopez A. (2019). Vital Signs: Surveillance for Acute Flaccid Myelitis—United States, 2018. MMWR Morb. Mortal. Wkly. Rep..

[32] Messacar K., Robinson C.C., Pretty K., Yuan J., Dominguez S.R. (2017). Surveillance for enterovirus D68 in colorado children reveals continued circulation. J. Clin. Virol..

[33] Srinivasan M., Niesen A., Storch G.A. (2018). Enterovirus D68 Surveillance, St. Louis, Missouri, USA, 2016. Emerg. Infect. Dis..

[34] Abedi G.R., Watson J.T., Nix W.A., Oberste M.S., Gerber S.I. (2018). Enterovirus and Parechovirus Surveillance—United States, 2014–2016. MMWR Morb. Mortal. Wkly. Rep..

[35] Messacar K., Pretty K., Reno S., Dominguez S.R. (2019). Continued biennial circulation of enterovirus D68 in Colorado. J. Clin. Virol..

[36] Uprety P., Curtis D., Elkan M., Fink J., Rajagopalan R., Zhao C., Bittinger K., Mitchell S., Ulloa E.R., Hopkins S. (2019). Association of Enterovirus D68 with Acute Flaccid Myelitis, Philadelphia, Pennsylvania, USA, 2009–2018. Emerg. Infect. Dis..

[37] Meijer A., Benschop K.S., Donker G.A., van der Avoort H.G. (2014). Continued seasonal circulation of enterovirus D68 in the Netherlands, 2011–2014. Eurosurveillance.

[38] Kramer R., Sabatier M., Wirth T., Pichon M., Lina B., Schuffenecker I., Josset L. (2018). Molecular diversity and biennial circulation of enterovirus D68: A systematic screening study in Lyon, France, 2010 to 2016. Eurosurveillance.

[39] Kreuter J.D., Barnes A., McCarthy J.E., Schwartzman J.D., Oberste M.S., Rhodes C.H., Modlin J.F., Wright P.F. (2011). A fatal central nervous system enterovirus 68 infection. Arch. Pathol. Lab. Med..

[40] Kirolos A., Mark K., Shetty J., Chinchankar N., Mcdougall C., Eunson P., Stevenson J., Templeton K. (2019). NHS Lothian EV-D68 Associated AFM Study Group; Pilley, E.; et al. Outcome of paediatric acute flaccid myelitis associated with enterovirus D68: A case series. Dev. Med. Child. Neurol..

[41] Knoester M., Helfferich J., Poelman R., Van Leer-Buter C., Brouwer O.F., Niesters H.G. (2019). Twenty-nine Cases of Enterovirus-D68-associated Acute Flaccid Myelitis in Europe 2016: A Case Series and Epidemiologic Overview. Pediatr. Infect. Dis. J..

[42] Messacar K., Schreiner T.L., Van Haren K., Yang M., Glaser C.A., Tyler K.L., Dominguez S.R. (2016). Acute flaccid myelitis: A clinical review of US cases 2012–2015. Ann. Neurol..

[43] Maloney J.A., Mirsky D.M., Messacar K., Dominguez S.R., Schreiner T., Stence N.V. (2015). MRI findings in children with acute flaccid paralysis and cranial nerve dysfunction occurring during the 2014 enterovirus D68 outbreak. AJNR Am. J. Neuroradiol..

[44] Christy A., Messacar K. (2019). Acute Flaccid Myelitis Associated With Enterovirus D68: A Review. J. Child Neurol..

[45] Hovden A.I., Pfeiffer H.C. (2015). Electrodiagnostic findings in acute flaccid myelitis related to enterovirus D68. Muscle Nerve.

[46] Yea C., Bitnun A., Robinson J., Mineyko A., Barton M., Mah J.K., Vajsar J., Richardson S., Licht C., Brophy J. (2017). Longitudinal Outcomes in the 2014 Acute Flaccid Paralysis Cluster in Canada. J. Child Neurol..

[47] Martin J.A., Messacar K., Yang M.L., Maloney J.A., Lindwall J., Carry T., Kenyon P., Sillau S.H., Oleszek J., Tyler K.L. (2017). Outcomes of Colorado children with acute flaccid myelitis at 1 year. Neurology.

[48] Hopkins S.E. (2017). Acute Flaccid Myelitis: Etiologic Challenges, Diagnostic and Management Considerations. Curr. Treat. Options Neurol..

[49] Center for Disease Control and Prevention (2018). Acute Flaccid Myelitis - Case Definitions. https://www.cdc.gov/acute-flaccid-myelitis/hcp/case-definition.html.

[50] Messacar K., Spence-Davizon E., Osborne C., Press C., Schreiner T.L., Martin J., Messer R., Maloney J., Burakoff A., Barnes M. (2019). Clinical Characteristics of Enterovirus A71 Neurologic Disease during an Outbreak in Children in Colorado, 2018. Lancet Infect. Dis..

[51] Center for Disease Control and Prevention (2019). Acute Flaccid Myelitis - AFM Confirmed U.S. Cases. https://www.cdc.gov/acute-flaccid-myelitis/afm-cases.html.

[52] Davies N.W., Brown L.J., Gonde J., Irish D., Robinson R.O., Swan A.V., Banatvala J., Howard R.S., Sharief M.K., Muir P. (2005). Factors influencing PCR detection of viruses in cerebrospinal fluid of patients with suspected CNS infections. J. Neurol. Neurosurg. Psychiatry.

[53] Center for Disease Control and Prevention (2019). Acute Flaccid Myelitis. https://www.cdc.gov/acute-flaccid-myelitis/index.html.

[54] Moline H., Kalaskar A., Pomputius W.F., Lopez A., Routh J., Kenyon C., Griffith J. (2019). Notes from the Field: Six Cases of Acute Flaccid Myelitis in Children—Minnesota, 2018. MMWR Morb. Mortal. Weekly Rep..

[55] Greninger A.L., Naccache S.N., Messacar K., Clayton A., Yu G., Somasekar S., Federman S., Stryke D., Anderson C., Yagi S. (2015). A novel outbreak enterovirus D68 strain associated with acute flaccid myelitis cases in the USA (2012–14): A retrospective cohort study. Lancet Infect. Dis..

[56] Sejvar J.J., Lopez A.S., Cortese M.M., Leshem E., Pastula D.M., Miller L., Glaser C., Kambhampati A., Shioda K., Aliabadi N. (2016). Acute Flaccid Myelitis in the United States, August-December 2014: Results of Nationwide Surveillance. Clin. Infect. Dis..

[57] Baltimore D. (1971). Expression of animal virus genomes. Bacteriol. Rev..

[58] Baggen J., Thibaut H.J., Strating J.R., van Kuppeveld F.J. (2018). The life cycle of non-polio enteroviruses and how to target it. Nat. Rev. Microbiol..

[59] Lulla V., Dinan A.M., Hosmillo M., Chaudhry Y., Sherry L., Irigoyen N., Nayak K.M., Stonehouse N.J., Zilbauer M., Goodfellow I. (2019). An upstream protein-coding region in enteroviruses modulates virus infection in gut epithelial cells. Nat. Microbiol..

[60] Imamura T., Fuji N., Suzuki A., Tamaki R., Saito M., Aniceto R., Galang H., Sombrero L., Lupisan S., Oshitani H. (2011). Enterovirus 68 among children with severe acute respiratory infection, the Philippines. Emerg. Infect. Dis..

[61] Kaida A., Kubo H., Sekiguchi J.I., Kohdera U., Togawa M., Shiomi M., Nishigaki T., Iritani N. (2011). Enterovirus 68 in children with acute respiratory tract infections, Osaka, Japan. Emerg. Infect. Dis..

[62] Lauinger I.L., Bible J.M., Halligan E.P., Aarons E.J., MacMahon E., Tong C.Y. (2012). Lineages, sub-lineages and variants of enterovirus 68 in recent outbreaks. PLoS ONE.

[63] Linsuwanon P., Puenpa J., Suwannakarn K., Auksornkitti V., Vichiwattana P., Korkong S., Theamboonlers A., Poovorawan Y. (2012). Molecular epidemiology and evolution of human enterovirus serotype 68 in Thailand, 2006–2011. PLoS ONE.

[64] Ikeda T., Mizuta K., Abiko C., Aoki Y., Itagaki T., Katsushima F., Katsushima Y., Matsuzaki Y., Fuji N., Imamura T. (2012). Acute respiratory infections due to enterovirus 68 in Yamagata, Japan between 2005 and 2010. Microbiol. Immunol..

[65] Meijer A., van der Sanden S., Snijders B.E., Jaramillo-Gutierrez G., Bont L., van der Ent C.K., Overduin P., Jenny S.L., Jusic E., van der Avoort H.G. (2012). Emergence and epidemic occurrence of enterovirus 68 respiratory infections in The Netherlands in 2010. Virology.

[66] Piralla A., Girello A., Grignani M., Gozalo-Margüello M., Marchi A., Marseglia G., Baldanti F. (2014). Phylogenetic characterization of enterovirus 68 strains in patients with respiratory syndromes in Italy. J. Med. Virol..

[67] Renois F., Bouin A., Andreoletti L. (2013). Enterovirus 68 in pediatric patients hospitalized for acute airway diseases. J. Clin. Microbiol..

[68] Opanda S.M., Wamunyokoli F., Khamadi S., Coldren R., Bulimo W.D. (2014). Genetic diversity of human enterovirus 68 strains isolated in Kenya using the hypervariable 3’-end of VP1 gene. PLoS ONE.

[69] Centers for Disease Control Prevention (2011). Clusters of acute respiratory illness associated with human enterovirus 68—Asia, Europe, and United States, 2008–2010. MMWR Morb. Mortal. Wkly. Rep..

[70] Gong Y.N., Yang S.L., Shih S.R., Huang Y.C., Chang P.Y., Huang C.G., Kao K.C., Hu H.C., Liu Y.C., Tsao K.C. (2016). Molecular evolution and the global reemergence of enterovirus D68 by genome-wide analysis. Medicine.

[71] Tan Y., Hassan F., Schuster J.E., Simenauer A., Selvarangan R., Halpin R.A., Lin X., Fedorova N., Stockwell T.B., Lam T.T. (2016). Molecular Evolution and Intraclade Recombination of Enterovirus D68 during the 2014 Outbreak in the United States. J. Virol..

[72] Du J., Zheng B., Zheng W., Li P., Kang J., Hou J., Markham R., Zhao K., Yu X.F. (2015). Analysis of Enterovirus 68 Strains from the 2014 North American Outbreak Reveals a New Clade, Indicating Viral Evolution. PLoS ONE.

[73] Pellegrinelli L., Giardina F., Lunghi G., Renteria S.C., Greco L., Fratini A., Galli C., Piralla A., Binda S., Pariani E. (2019). Emergence of divergent enterovirus (EV) D68 sub-clade D1 strains, northern Italy, September to October 2018. Eurosurveillance.

[74] Yip C.C.Y., Lo J., Sridhar S., Lung D., Luk S., Chan K.H., Chan J., Cheng V., Woo P., Yuen K.Y. (2017). First Report of a Fatal Case Associated with EV-D68 Infection in Hong Kong and Emergence of an Interclade Recombinant in China Revealed by Genome Analysis. Int. J. Mol. Sci..

[75] Rohll J.B., Percy N., Ley R., Evans D.J., Almond J.W., Barclay W.S. (1994). The 5’-untranslated regions of picornavirus RNAs contain independent functional domains essential for RNA replication and translation. J. Virol..

[76] Yeh M.T., Wang S.W., Yu C.K., Lin K.H., Lei H.Y., Su I.J., Wang J.R. (2011). A single nucleotide in stem loop II of 5’-untranslated region contributes to virulence of enterovirus 71 in mice. PLoS ONE.

[77] De Jesus N., Franco D., Paul A., Wimmer E., Cello J. (2005). Mutation of a single conserved nucleotide between the cloverleaf and internal ribosome entry site attenuates poliovirus neurovirulence. J. Virol..

[78] Yuan L., Huang X.Y., Liu Z.Y., Zhang F., Zhu X.L., Yu J.Y., Ji X., Xu Y.P., Li G., Li C. (2017). A single mutation in the prM protein of Zika virus contributes to fetal microcephaly. Science.

[79] Zhang Y., Cao J., Zhang S., Lee A.J., Sun G., Larsen C.N., Zhao H., Gu Z., He S., Klem E.B. (2016). Genetic changes found in a distinct clade of Enterovirus D68 associated with paralysis during the 2014 outbreak. Virus Evol..

[80] Dyrdak R., Grabbe M., Hammas B., Ekwall J., Hansson K.E., Luthander J., Naucler P., Reinius H., Rotzén-Östlund M., Albert J. (2016). Outbreak of enterovirus D68 of the new B3 lineage in Stockholm, Sweden, August to September 2016. Eurosurveillance.

[81] Chen I.J., Hu S.C., Hung K.L., Lo C.W. (2018). Acute flaccid myelitis associated with enterovirus D68 infection: A case report. Medicine.

[82] Piralla A., Principi N., Ruggiero L., Girello A., Giardina F., De Sando E., Caimmi S., Bianchini S., Marseglia G.L., Lunghi G. (2018). Enterovirus-D68 (EV-D68) in pediatric patients with respiratory infection: The circulation of a new B3 clade in Italy. J. Clin. Virol..

[83] Wang G., Zhuge J., Huang W., Nolan S.M., Gilrane V.L., Yin C., Dimitrova N., Fallon J.T. (2017). Enterovirus D68 Subclade B3 Strain Circulating and Causing an Outbreak in the United States in 2016. Sci. Rep..

[84] Esposito S., Chidini G., Cinnante C., Napolitano L., Giannini A., Terranova L., Niesters H., Principi N., Calderini E. (2017). Acute flaccid myelitis associated with enterovirus-D68 infection in an otherwise healthy child. Virol. J..

[85] Knoester M., Schölvinck E.H., Poelman R., Smit S., Vermont C.L., Niesters H.G., Van Leer-Buter C.C. (2017). Upsurge of Enterovirus D68, the Netherlands, 2016. Emerg. Infect. Dis..

[86] Carballo C.M., Erro M.G., Sordelli N., Vazquez G., Mistchenko A.S., Cejas C., Rodriguez M., Cisterna D.M., Freire M.C., Contrini M.M. (2019). Acute Flaccid Myelitis Associated with Enterovirus D68 in Children, Argentina, 2016. Emerg. Infect. Dis..

[87] Bal A., Sabatier M., Wirth T., Coste-Burel M., Lazrek M., Stefic K., Brengel-Pesce K., Morfin F., Lina B., Schuffenecker I. (2019). Emergence of enterovirus D68 clade D1, France, August to November 2018. Eurosurveillance.

[88] Center for Disease Control and Prevention Specimen Collection, Storage, & Shipment. https://www.cdc.gov/non-polio-enterovirus/lab-testing/specimen-collection.html.

[89] Harvala H., Broberg E., Benschop K., Berginc N., Ladhani S., Susi P., Christiansen C., McKenna J., Allen D., Makiello P. (2018). Recommendations for enterovirus diagnostics and characterisation within and beyond Europe. J. Clin. Virol..

[90] Hixon A.M., Yu G., Leser J.S., Yagi S., Clarke P., Chiu C.Y., Tyler K.L. (2017). A mouse model of paralytic myelitis caused by enterovirus D68. PLoS Pathog..

[91] Morrey J.D., Wang H., Hurst B., Zukor K., Siddharthan V., Van Wettere A., Sinex D., Tarbet E. (2018). Causation of Acute Flaccid Paralysis by Myelitis and Myositis in Enterovirus-D68 Infected Mice Deficient in Interferon alphabeta/gamma Receptor Deficient Mice. Viruses.

[92] Brown D.M., Hixon A.M., Oldfield L.M., Zhang Y., Novotny M., Wang W., Das S.R., Shabman R.S., Tyler K.L., Scheuermann R.H. (2018). Contemporary Circulating Enterovirus D68 Strains Have Acquired the Capacity for Viral Entry and Replication in Human Neuronal Cells. MBio.

[93] Sun S., Bian L., Gao F., Du R., Hu Y., Fu Y., Su Y., Wu X., Mao Q., Liang Z. (2019). A neonatal mouse model of Enterovirus D68 infection induces both interstitial pneumonia and acute flaccid myelitis. Antivir. Res..

[94] Zhang C., Zhang X., Dai W., Liu Q., Xiong P., Wang S., Geng L., Gong S., Huang Z. (2018). A Mouse Model of Enterovirus D68 Infection for Assessment of the Efficacy of Inactivated Vaccine. Viruses.

[95] Hixon A.M., Clarke P., Tyler K.L. (2017). Evaluating Treatment Efficacy in a Mouse Model of Enterovirus D68-Associated Paralytic Myelitis. J. Infect. Dis..

[96] Tyler K.L., McPhee D.A., Fields B.N. (1986). Distinct pathways of viral spread in the host determined by reovirus S1 gene segment. Science.

[97] Sun J., Hu X.Y., Yu X.F. (2019). Current Understanding of Human Enterovirus D68. Viruses.

[98] Dai W., Zhang C., Zhang X., Xiong P., Liu Q., Gong S., Geng L., Zhou D., Huang Z. (2018). A virus-like particle vaccine confers protection against enterovirus D68 lethal challenge in mice. Vaccine.

[99] Harrison C.J., Weldon W.C., Pahud B.A., Jackson M.A., Oberste M.S., Selvarangan R. (2019). Neutralizing Antibody against Enterovirus D68 in Children and Adults before 2014 Outbreak, Kansas City, Missouri, USA(1). Emerg. Infect. Dis..

[100] Zhang Y., Moore D.D., Nix W.A., Oberste M.S., Weldon W.C. (2015). Neutralization of Enterovirus D68 isolated from the 2014 US outbreak by commercial intravenous immune globulin products. J. Clin. Virol..

[101] Royston L., Essaidi-Laziosi M., Pérez-Rodríguez F.J., Piuz I., Geiser J., Krause K.H., Huang S., Constant S., Kaiser L., Garcin D. (2018). Viral chimeras decrypt the role of enterovirus capsid proteins in viral tropism, acid sensitivity and optimal growth temperature. PLoS Pathog..

[102] Hixon A.M., Clarke P., Tyler K.L. (2019). Contemporary circulating enterovirus D68 strains infect and undergo retrograde axonal transport in spinal motor neurons independent of sialic acid. J. Virol..

[103] Walther T., Karamanska R., Chan R.W., Chan M.C., Jia N., Air G., Hopton C., Wong M.P., Dell A., Peiris J.M. (2013). Glycomic analysis of human respiratory tract tissues and correlation with influenza virus infection. PLoS Pathog..

[104] Baggen J., Thibaut H.J., Staring J., Jae L.T., Liu Y., Guo H., Slager J.J., de Bruin J.W., van Vliet A.L., Blomen V.A. (2016). Enterovirus D68 receptor requirements unveiled by haploid genetics. Proc. Natl. Acad. Sci. USA.

[105] Liu Y., Sheng J., Baggen J., Meng G., Xiao C., Thibaut H.J., Van Kuppeveld F.J., Rossmann M.G. (2015). Sialic acid-dependent cell entry of human enterovirus D68. Nat. Commun..

[106] Wei W., Guo H., Chang J., Yu Y., Liu G., Zhang N., Willard S.H., Zheng S., Yu X.F. (2016). ICAM-5/Telencephalin Is a Functional Entry Receptor for Enterovirus D68. Cell Host Microbe.

[107] Kolatkar P.R., Bella J., Olson N.H., Bator C.M., Baker T.S., Rossmann M.G. (1999). Structural studies of two rhinovirus serotypes complexed with fragments of their cellular receptor. EMBO J..

[108] Xiao C., Bator C.M., Bowman V.D., Rieder E., He Y., Hébert B., Bella J., Baker T.S., Wimmer E., Kuhn R.J. (2001). Interaction of coxsackievirus A21 with its cellular receptor, ICAM-1. J. Virol..

